# Constraints to estimating the prevalence of trypanosome infections in East African zebu cattle

**DOI:** 10.1186/1756-3305-3-82

**Published:** 2010-09-06

**Authors:** Andrew P Cox, Olga Tosas, Aimee Tilley, Kim Picozzi, Paul Coleman, Geoff Hide, Susan C Welburn

**Affiliations:** 1 Centre for Infectious Diseases, School of Biomedical Sciences, College of Medicine and Veterinary Medicine, University of Edinburgh, 1 Summerhall Square, Edinburgh, EH9 1QH, UK; 2 Centre for Parasitology and Disease Research, School of Environment and Life Sciences, University of Salford, The Crescent, Salford, Manchester, M5 4WT, UK; 3 London School of Hygiene and Tropical Medicine, Keppel Street, London, WC1E 7HT, UK

## Abstract

**Background:**

In East Africa, animal trypanosomiasis is caused by many tsetse transmitted protozoan parasites including *Trypanosoma vivax*, *T. congolense *and subspecies of *T. brucei *s.l. (*T. b. brucei *and zoonotic human infective *T. b. rhodesiense*) that may co-circulate in domestic and wild animals. Accurate species-specific prevalence measurements of these parasites in animal populations are complicated by mixed infections of trypanosomes within individual hosts, low parasite densities and difficulties in conducting field studies. Many Polymerase Chain Reaction (PCR) based diagnostic tools are available to characterise and quantify infection in animals. These are important for assessing the contribution of infections in animal reservoirs and the risk posed to humans from zoonotic trypanosome species. New matrices for DNA capture have simplified large scale field PCR analyses but few studies have examined the impact of these techniques on prevalence estimations.

**Results:**

The Whatman FTA matrix has been evaluated using a random sample of 35 village zebu cattle from a population naturally exposed to trypanosome infection. Using a generic trypanosome-specific PCR, prevalence was systematically evaluated. Multiple PCR samples taken from single FTA cards demonstrated that a single punch from an FTA card is not sufficient to confirm the infectivity status of an individual animal as parasite DNA is unevenly distributed across the card. At low parasite densities in the host, this stochastic sampling effect results in underestimation of prevalence based on single punch PCR testing. Repeated testing increased the estimated prevalence of all *Trypanosoma *spp. from 9.7% to 86%. Using repeat testing, a very high prevalence of pathogenic trypanosomes was detected in these local village cattle: *T. brucei *(34.3%), *T. congolense *(42.9%) and *T. vivax *(22.9%).

**Conclusions:**

These results show that, despite the convenience of Whatman FTA cards and specific PCR based detection tools, the chronically low parasitaemias in indigenous African zebu cattle make it difficult to establish true prevalence. Although this study specifically applies to FTA cards, a similar effect would be experienced with other approaches using blood samples containing low parasite densities. For example, using blood film microscopy or PCR detection from liquid samples where the probability of detecting a parasite or DNA molecule, in the required number of fields of view or PCR reaction, is less than one.

## Background

Animal trypanosomiasis, or 'nagana', is an infectious disease of livestock caused by a range of protozoan parasites of the genus *Trypanosoma*. In Africa, *Trypanosoma vivax, Trypanosoma congolense *and *Trypansoma brucei *s.l. are the three most important species of trypanosomes responsible for considerable production losses and livestock morbidity where they occur [1,2]. All three species are transmitted by tsetse flies in the genus *Glossina*, in which they have obligate life cycle stages. *Trypanosoma brucei *s.l. comprises three sub species: *Trypanosoma brucei gambiense *and *Trypanosoma brucei rhodesiense *are human infective variants that cause the West African chronic form of sleeping sickness and the East African acute form of sleeping sickness, respectively [3], while *Trypanosoma brucei brucei *does not infect humans and is mildly pathogenic in cattle [4]. A fourth species *Trypanosoma theileri *is usually non pathogenic but commonly found in cattle worldwide [5-7].

In Uganda and other parts of East Africa, *T. b. brucei *and *T. b. rhodesiense *co-circulate in cattle, other livestock and wild animal species. Outbreaks of human infection occur periodically [8,9], and cattle have been shown to play a key role in the generation of human sleeping sickness epidemics in Uganda [3,10-13]. Understanding the epidemiology of *T. brucei *s.l. in cattle is important both for understanding and controlling animal trypanosomiasis as well as for estimating the size of the reservoir of human infective parasites and planning appropriate public health control measure [3,13].

For determination of trypanosome infection status in rural African settings, microscopy-based techniques using direct observation of wet blood films, microscopic examination of Giemsa stained blood smears, or concentration techniques such as the Buffy Coat Technique (BCT) and the Haematocrit Centrifugation Technique (HCT) are the most common methods of parasite detection, and have been long considered the best diagnostic methods available [14]. Molecular diagnostic tools, and in particular PCR, have improved the detection of trypanosome infections over standard parasitological techniques, by lowering the parasitaemia detection limit by several orders of magnitude. PCR has offered the promise of more sensitive detection and the ability to detect and differentiate all trypanosome species using either a series of specific single PCR methods [15-17] or single methods which can detect multiple species [18-21].

Comparative studies show that microscopy has a very poor sensitivity compared to diagnosis with molecular tools, suggesting that previous studies using standard parasitological methods may have significantly underestimated both animal- and herd-level prevalence of these pathogens [14]. Reported analytical sensitivity of microscopy ranges between detectable levels of parasitaemia of between 2.5 x10^2 ^to 5 x10^3 ^parasites/ml of blood (applying concentration methods, such as the HCT or buffy coat technique BCT) but this is highly contingent on trypanosome species [14]. Under optimal laboratory conditions using highly purified DNA, PCR based methods have been reported to detect the presence of parasite DNA equivalent to a single trypanosome in 10 ml host blood [22]. The analytic detection limit of the *T. brucei *s.l. specific PCR has been shown to be as low as 1/10 of the genetic material of a single trypanosome template per PCR reaction [23]. PCR, based on the ribosomal intergenic transcribed spacer (ITS) regions, is able to detect trypanosome DNA at a dilution equivalent to less than one parasite/ml of host blood [19]. Furthermore, primers specifically designed to target particular identifying DNA sequences ensure high species-specificity of PCR, removing the ambiguity of unreliable identification of trypanosome species by microscopy.

Field applications of PCR include estimating trypanosome prevalence for the monitoring of control programmes, though due to the cost and level of laboratory equipment involved, PCR is currently not suitable for diagnostic testing of individual animals for treatment decisions at the local level. PCR based methods are invaluable for addressing important epidemiological questions regarding the zoonotic potential of *T. brucei *s.l. PCR has therefore become the diagnostic tool of choice for a number of studies investigating the epidemiology of trypanosomiasis, especially since advances in preservation methodology for biological samples have facilitated collection and stabilization of field samples of sufficiently high quality for molecular analysis [3,14,24].

However, using these new molecular approaches, the prevalence of trypanosomes in naturally infected cattle has often been found to be very low, at levels similar to those estimated with microscopy detection [14,25]. This begs the question of whether this is either a real phenomenon or one that is generated by the lack of sensitivity in the detection systems used (i.e. arising from issues associated with the sampling procedure, the sample-storage matrix, the PCR method, or a combination of these parameters).

PCR primers inherently exhibit high target-specificity but the sensitivities of the test systems when applied to field samples are often lower than expected considering the detection limit of the PCRs themselves [26]. Several factors may contribute to the lower than expected sensitivity including competing DNA target and residual PCR inhibitors in the test material [27,28]. Differences in sensitivity between PCR methods may be attributable to a higher number of copies of the target sequence for the *T. brucei *s.l. specific PCR (10,000 copies/genome) as compared to the ITS-PCR (200 copies/genome) [23,29]. It may also be that the efficiency of PCR amplifications from the FTA filter paper matrix depend on the target sequence length (1250 base pairs for the ITS-PCR and 173 for *the T. brucei *s.l. specific PCR [29]).

However, in this paper we examine for the first time the possibility that the observed low prevalence in cattle from a trypanosome-endemic setting estimated using PCR methods is more straightforwardly explained as essentially a function of the sample-storage element of the testing system - the FTA card matrix.

FTA card matrices preserve the DNA in the biological sample by lysing the cells and fixing DNA *in situ *to the filter-paper matrix. It is standard practice to take a single small punch from the FTA card for PCR analysis. The sample volume contained on the punched-out material tends to represent only a small fraction (often < 1%) of the total blood sample captured on the FTA card, which is itself extremely small in relation to the volume of blood within a host. For PCR based applications the assayed volume may be typically only a single microlitre. This raises the possibility that punch samples taken from FTA cards for PCR-testing may result in an underestimation of the prevalence in the host population, particularly when that population comprises individuals with very low parasitaemias.

Here we have tested the hypothesis that parasite DNA contained in a blood sample may be localized (i.e. unevenly distributed or clustered) on the FTA card matrix, with the result that taking a single punch as a template for a PCR-based diagnostic test may result in a false negative result simply because that punch of blood selected did not include any parasite DNA. Moreover, the likelihood of any single punch giving a false-positive is inversely related to the parasitaemia (i.e. parasite density) in the host animal blood.

We evaluated this hypothesis by conducting exhaustive, multiple ITS-PCR testing of FTA cards containing blood taken from an indigenous population of cattle from a single village in Uganda that is naturally exposed to trypanosome infections. The relationship between underestimation of prevalence and FTA card sub-sampling was further examined using an artificial dilution series, containing trypanosomes diluted in cow blood. The findings have important implications for the design of PCR-based detection systems for the estimation of trypanosome prevalence, and our understanding of trypanosome epidemiology.

## Materials and methods

### Sample collection

Blood samples were collected from 35 zebu cattle in the village of Ojilai, Tororo in Uganda in June 2001 as part of a routine sampling protocol from a larger longitudinal study [30]. Approximately 200 μl of blood from the ear vein of each cow was applied to Whatman FTA™ cards (Whatman, Maidstone, Kent, UK) and allowed to dry for a minimum of twenty-four hours at room temperature prior to long term storage, again at room temperature, an established method of preservation for sensitive detection of trypanosome infections by PCR [14].

### Sample preparation and PCR amplification of DNA

All blood samples were analysed by ITS PCR according to established protocols [19]. ITS PCR targets the internal transcribed spacers (ITS) located within the ribosomal RNA genes (200 copies/genome) and discriminates between the important pathogenic African trypanosome species affecting livestock, including *Trypanosoma brucei *s.l. [19]. For each PCR reaction one 3 mm punch was cut from the samples on the Whatman FTA^® ^Card and processed according to the manufacturers instructions. Once dried, the discs were transferred to PCR tubes to seed the reactions. One positive control (genomic DNA) and two negative controls (blank FTA disc; disc containing uninfected bovine DNA) were run with each set of reactions.

### Mapping of PCR results

Each blood sample applied to the FTA card was subject to between 92 and 114 individual PCR assays (depending on the amount of blood available on the sample). The position of each sample punch taken from the FTA card was recorded so that a positive result could be traced to the position on the card from which the sample punch was originally taken. The total number of trypanosome positive and trypanosome negative punches was recorded for each animal sample. For each FTA card, the number of positives for each trypanosome species was also recorded.

### Preparation of controls

Uninfected bovine blood (UK origin) was used as a negative control to ensure that results were not biased by false positives during repeated PCR assays. A positive control sample was constructed with known numbers of *Trypanosoma b. brucei *trypanosomes (insect form procyclics) diluted in whole cow blood (UK origin). The resultant concentration of trypanosomes was calculated with allowance for the dilution factor, at 508 trypanosomes per millilitre using a mean of thirty readings using a Neubauer haemocytometer. Positive and negative controls were treated in an identical manner to the test samples derived from zebu cattle (see Table 1).

**Table 1:** Results of multiple PCRs on zebu cattle blood samples

Sample No.	*T. theileri*	*T. brucei*	*T. congolense*	*T. vivax*	Negative
1	12	0	0	0	80
2	2	0	0	0	101
3	6	3	7	0	85
4	1	1	0	0	98
5	0	0	0	0	109
6	2	2	4	2	88
7	4	0	0	0	100
8	8	7	4	0	91
9	8	0	0	13	83
10	7	0	2	0	92
11	3	0	0	1	106
12	10	2	0	3	87
13	0	0	0	0	110
14	2	1	3	0	100
15	21	10	6	0	65
16	6	0	3	0	96
17	12	0	0	0	90
18	1	0	1	0	98
19	3	0	3	2	94
20	3	0	0	0	100
21	19	0	0	0	85
22	18	1	2	0	78
23	0	0	0	0	102
24	0	0	0	0	107
25	3	0	3	0	95
26	4	1	2	0	101
27	0	0	0	0	98
28	4	0	0	0	97
29	1	1	0	0	100
30	1	0	3	3	95
31	1	1	0	1	100
32	15	0	0	0	99
33	2	0	0	0	100
34	9	3	14	14	73
35	2	0	1	0	99

Negative control	0	0	0	0	107
Positive control	0	45	0	0	101

Results obtained from multiple PCR of thirty five blood samples from zebu cattle. The frequency of positive results for *T. theileri, T. brucei, T. congolense, T. vivax *and of negative results is recorded in columns 2 to 6 respectively.

### Experimental dilution series of trypanosomes

To examine the effect of parasite density on PCR detection, an artificial dilution series was prepared, containing trypanosomes diluted in cow blood at a concentration of 10^6 ^trypanosomes per ml. *Trypanosoma brucei brucei*, strain Buteba135 [31,32] cultured procyclic trypanosomes were diluted in bovine blood as described above. A tenfold series of dilutions were prepared giving three dilution series: 10^-5^, 10^-6 ^and 10^-7 ^of the original stock (equivalent to 10, 1 and 0.1 trypanosomes per ml). The dilutions were then placed on Whatman FTA cards and allowed to air dry before PCR analysis. Each sample was tested using ITS-PCR [19] eight times at each level dilution point across the series.

## Results

### Experimental dilution series of trypanosomes

**Figure 1:**
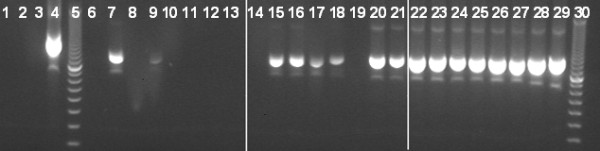
**Success rate of detection of trypanosomes in the artificial dilution series of *T. b. brucei***. Cultured *T. brucei brucei *was diluted in cow blood at a concentration of 10^6 ^trypanosomes per millilitre and placed onto Whatman FTA cards. Lanes 1 to 3 are negative controls, lane 4 is a positive control lane 5 is a DNA marker, lanes 6 to 13 show the results of repeated PCR of a 10^-7 ^dilution of the original stock (equivalent to 0.1 trypanosome per ml). Lanes 14 to 21 show the results of repeated PCR of a 10^-6 ^dilution of the original stock (equivalent to 1 trypanosome per ml). Lanes 22 to 29 show the results of repeated PCR of a 10^-5 ^dilution of the original stock (equivalent to 10 trypanosomes per ml). Lane 30 is a DNA marker.

The results of PCR reaction series performed on the artificial dilution series created from a starting concentration of 10^6 ^trypanosomes per millilitre (Figure 1) show that at low dilutions (i.e. high parasite densities) ITS detection of parasite DNA occurred in 100% of assays. However, at 10 and 100 fold lower parasite densities, success of ITS detection of parasite DNA reduced to 75% and 25%, respectively.

### Trypanosome detection in naturally infected zebu cattle

**Figure 2:**
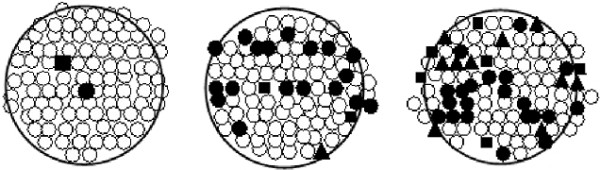
**Mapping of positive PCR punches on FTA cards**. The figure shows three diagrammatic representations of the repeated PCR of blood samples from zebu cattle. Each small circle or shape represents a punch taken for PCR analysis. The positions of each punch were recorded and the results for that PCR were related back to the position on the original sample. Key; open circle, negative PCR result; closed circle, *T. theileri*; closed triangle, *T. brucei*; closed square, *T. congolense*. Examples of a low, medium and high parasitaemia result are shown.

In total, 3622 PCR reactions were undertaken from the 35 FTA blood spot samples (an average of 103.4 PCR reactions per card, range 92 - 114). All four species of *Trypanosoma *were detected in this cohort. The diagnostic result and position of positive trypanosome PCR amplifications on the FTA paper were recorded for each individual PCR result. Figure 2 shows an example of the results, with animals representing low, medium and high PCR-test results, which we assume reflects relative trypanosome parasite density. This demonstrates the role of chance in determining whether a PCR-test performed on a single punch taken from the blood spot gives a positive or negative result and in determining the correct species of trypanosome as the cause of infection. Examination of all the PCR amplifications from each animal showed only five (14.2%) animals that were consistently PCR-negative for any trypanosome infection and 14 (40%) consistently PCR-negative for any of the three pathogenic species. Mixed infections were observed in 60% (n = 21) of the animals.

The non-pathogenic *Trypanosoma theileri *was most frequently detected and displayed the highest PCR-positive density, giving a prevalence of 85.7% (n = 30). The pathogenic species *Trypanosoma brucei*, *Trypanosoma congolense *and *Trypanosoma vivax *showed lower levels of PCR-positive density, with 60% (n = 21) of cows positive for at least one of the pathogenic trypanosome species. The prevalence of the individual pathogenic species were: *Trypanosoma brucei*, 34.3% (n = 12); *Trypanosoma congolense*, 42.9% (n = 15) and *Trypanosoma vivax*, 22.9% (n = 8). The negative control samples from cattle of U.K. origin remained negative throughout.

### Single versus multiple PCR tests

**Figure 3:**
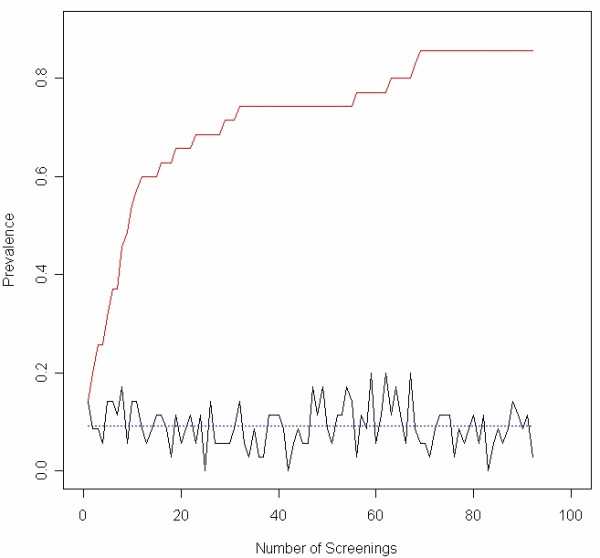
**Cumulative prevalence achieved at each round of screening of blood samples taken from thirty-five African zebu cattle**. The figure shows the plot of the cumulative prevalence (upper curve) for all species of trypanosome at each round of screening of the thirty five blood samples. As the number of screenings increases the cumulative prevalence also continues to increase as new samples are found positive. The cross sectional prevalence at each round of screening is also shown (lower curve). The mean cross sectional prevalence across all screenings is shown by the dotted line (9.7%).

A comparison of the impact of screening a single punch versus exhaustive punch replicates (cumulative prevalence) across the 35 cattle samples is shown in Table 2 and illustrated in Figure 3. The prevalence for each trypanosome species is significantly increased following repeated testing. Infection with any trypanosome species is common and overall infection with any species increases from an average of 9.7% with a single punch selected at random from the blood spot to 85.7% using the total cumulative prevalence. For *T. brucei *infection the prevalence in this cohort increases from an average of 0.91% with a single punch to 34.3% when using the total cumulative prevalence (Table 2). Using the data collected and making assumptions of mono-dispersion of parasites in host blood, we estimate that a minimum of 950 trypanosomes per ml of blood is the threshold to be 95% certain that we will detect an infection with a single punch.

**Table 2:** Prevalence of trypanosome species in zebu cattle

Species	Single PCR per Sample Average Prevalence (%)	Cumulative Prevalence (%)
*T. theileri*	5.26	85.7 (69.7 - 95.2)
*T. brucei*	0.91	34.3 (19.1 - 52.2)
*T. congolense*	1.58	42.9 (26.3 - 60.6)
*T vivax*	1.05	22.9 (10.4 - 40.1)
Any trypanosomes	9.7	85.71 (69.7 - 95.2)
Mixed Infections (All Samples)	0	60 (42.1 - 76.1)

The table shows (in the first column) the prevalence of the different species of trypanosomes and the prevalence of mixed infections detected in thirty-five blood samples collected from zebu cattle obtained from a single punch selected at random from the FTA card per animal. This would have been the prevalence assumed on average in an epidemiological study. In the second column the cumulative prevalence of the different species of trypanosomes and the prevalence of mixed infections is shown based on between 92 and 114 punches. 95% confidence intervals are shown in brackets. The mean diagnosed prevalence of any trypanosome species for all repeat screenings was 9.7%.

## Discussion

These experiments demonstrate two things. Firstly, parasite DNA sits where it is placed on the card and does not spread evenly across the matrix. Secondly, single punch, PCR sampling from FTA cards cannot be used to accurately measure the prevalence of either any or all trypanosome species in cattle populations; critically the level of underestimation using a single punch PCR test will tend increase when parasite densities are low (as typically found in natural trypanosome infections of indigenous cattle). Therefore, a higher prevalence of pathogenic trypanosomes (*T. brucei*, *T. congolense *and *T. vivax*) may be circulating in naturally infected village zebu cattle than previously estimated.

Classically, most field studies of African trypanosomiasis have approached the analysis and collection of samples in a similar way. A large number of samples are collected and subjected to a single diagnostic test for presence or absence of a particular species of trypanosome [33-35]. Such studies are challenging logistically and the widespread availability and affordability of FTA cards and availability of DNA based methods has considerably improved the feasibility of large-scale epidemiological studies. FTA cards are a convenient matrix for field samples and have the advantage that they can be stored for subsequent analysis. Data are derived, typically from the application of a single diagnostic test to calculate a prevalence value for each species of trypanosome(s) and the raw data may then be used for statistical analysis or disease modelling.

It is evident from the results presented here that data derived from studies where a single sample (punch) is used, as the basis for a diagnostic PCR test will considerably underestimate the prevalence of trypanosomes within a population of hosts. This is not a function of the sensitivity of the PCR technique, but due to the probabilistic effect of detecting trypanosome DNA in the particular aliquot of blood that was selected for analysis from the FTA card. This is particularly critical when parasite density is very low and the probability of a punch containing a trypanosome (or trypanosome DNA) is correspondingly low.

Here we have experimentally demonstrated a relationship between parasite density and the resulting prevalence obtained. The challenge is to be able to obtain the true prevalence in a sample taken from an FTA matrix, irrespective of parasite density. This raises the question as to how many tests must be undertaken to be confident of the prevalence value obtained. In this study of 35 naturally infected animals, more than 90 replicates were required but this will depend upon the density of the natural infections in the host population. Such extensive and in depth analysis as described here may not be practical for widespread screening but it does provide valuable insights that may help inform sampling strategies. These results help us understand the impact of low parasite densities in host samples on PCR based epidemiological screening methods. Further work needs to develop practical techniques or mathematical approaches to enable us to infer the 'true' underlying prevalence from a single diagnostic event.

The problems in applying new molecular methods and using new sampling strategies and tools are not simply those of the sensitivity of the PCR test. The use of real time PCR to quantify the DNA in a single sample can be both highly sensitive and quantitative [36] but will be subject to the same basic stochastic phenomena shown in this study, the result being entirely dependent on parasite density. Whole genome amplification [23] to enrich DNA from a solution of DNA derived from FTA cards or elution of total DNA from cards may minimise this stochastic effect, but there remains an absolute requirement with all DNA based methods to obtain sufficient target pathogen DNA within the aliquot of sample drawn for assay. In each case the assayed volume of blood is tiny in comparison to the volume of blood that can potentially contain trypanosomes and this is likely to be exacerbated if trypanosomes tend to be aggregated within the host. One solution may be to apply a model that enables prediction of prevalence from a limited repeat sampling strategy.

That 60% of the cattle samples examined were found to be positive for pathogenic trypanosomes after repeated analysis in this study, exhibiting very low levels of parasitaemia, suggests that the majority of natural infections may be asymptomatic and that a high proportion of the animals act as carriers of one or more parasites that can be pathogenic to other species. This has important implications for our understanding of the epidemiology of trypanosomiasis and how the disease may be diagnosed and controlled, particularly for human sleeping sickness in which the cumulative prevalence for *T. brucei *was 34.3%, much higher than is usually reported and may represent underestimation in previous studies. Since it is estimated that the human infective *Trypanosoma brucei rhodesiense *exists in *T. brucei *populations at a proportion of around 33% [37] many of these animals may be acting as carriers of the human infective sub species. The high levels of *T. brucei *found in zebu cattle have the potential to be very important animal reservoirs for human disease. Previous studies may have initially under-estimated the scale of the *T. brucei *s.l. reservoir in different livestock species, and may consequently have under-estimated the impact that mass treatment activities have had in addressing it. Regarding the potential reservoir of zoonotic *T. b. rhodesiense*, cattle and pigs may be a more substantial risk than previously estimated highlighting the need to specifically consider the test parameters for PCR protocols [38] in future studies.

The results presented in this paper have wider implications. PCR detection systems are now commonly used for the detection of sleeping sickness in humans and animal trypanosomiasis. Diagnosis of the *T. brucei gambiense *form of HAT has always proven difficult due to disagreement on infection status between diagnostic techniques, and current methods involve a complex algorithm of sequential diagnostic tests [39]. Accurate measurement of prevalence is important not only in understanding the scale of human and animal disease but also in determining the role of animal reservoirs in human disease. Accurate measurement of trypanosome infection in the tsetse fly vector is also important and many studies make use of FTA cards for the collection of DNA from tsetse [e.g. [40,41]]. In order to better understand the epidemiology of the parasite more attention should be given to the distribution of the parasite in the population in a addition to the level of infected/uninfected host. In a wider context, the results reported here may be applicable to a wide range of parasitic diseases, for example malaria, where low parasite densities may mask the distribution of the disease. Future research should, of course, be directed at the development of more sensitive and specific diagnostic tools for use on low parasite density infections found in natural populations within the field. But alongside this, there is a need for tools for data interpretation that take into account for the stochastic nature of the sampling process in low parasite density infections.

Finally, it is imperative to standardise protocols or establish, as we do here, the relative performance of different protocols across study populations and between testing centres, in order to make meaningful comparisons between different studies.

## Competing interests

The authors declare that they have no competing interests.

## Authors' contributions

APC carried out the molecular genetic analyses, participated in the data analysis, was involved in field collection and drafted the manuscript. OT participated in the design of the study, participated in the statistical analysis and helped to draft the manuscript. AT participated in the design of the study and helped to carry out the molecular genetic analyses. PC participated in the study design, helped with the statistics and helped to draft the manuscript. GH helped to conceive the study, participated in its design, helped with the design of the molecular analyses, helped coordination of the study, assisted in obtaining funding (University of Salford) and helped to draft the manuscript. KP and SCW helped to conceive the study, participated in its design, were involved in fieldwork, helped coordination of the study, assisted in obtaining funding (DFID) and helped to draft the manuscript. All authors read and approved the final manuscript.
